# Phylogenetic analysis of *Cyprinus acutidorsalis* (Wang, 1979) from the Hainan population using complete mitochondrial genome

**DOI:** 10.1080/23802359.2024.2323004

**Published:** 2024-06-24

**Authors:** Qingfeng Zhang, Fangyuan Li, Yang Dong, Xingwei Cai, Yuan Gu, Zhenjiang Wang, Zhixin Shen

**Affiliations:** a Hainan Academy of Ocean and Fisheries Sciences, Haikou, China; b Research Center for Freshwater Bioresource and Eco-environment Protection in Hainan Province, Haikou, China

**Keywords:** Mitogenome, *Cyprinus* species, phylogenetic position, Hainan population

## Abstract

*Cyprinus acutidorsalis* (Wang, 1979) is an endemic fish in China that is sparsely distributed in the Hainan provinces and Guangxi Zhuang Autonomous Region (GZAR). In this study, the complete mitochondrial genome of *C. acutidorsalis* from the Hainan population from the Wanquan River was sequenced, and its phylogenetic relationship was analyzed. The circular mtDNA was 16,581 bp in length, and the overall base composition was A (32.0%), C (27.5%), T (24.8%), and G (15.70%), with a slight bias toward A + T. The complete mitogenome encoded 13 protein-coding genes (PCGs), 22 tRNA genes, two rRNA genes, and a control region. Phylogenetic analysis indicated that the most closely related fish to *C. acutidorsalis* from the Hainan population was *C. acutidorsalis* from the Guangxi population. These findings offer basic molecular data and a better understanding of the phylogenetic relationships among the *Cyprinus* species.

## Introduction

*Cyprinus acutidorsalis* (Wang, 1979), a species of *Cyprinus* in the Cyprinidae family, is a salty freshwater fish that is sparsely distributed in the Hainan Wanquan and Guangxi Qinjiang rivers (Yao et al. 2014). The phylogenetic relationships in the genus *Cyprinus* are complex. In this study, the complete mitochondrial genome of *C. acutidorsalis* from the Hainan population was determined to provide basic molecular data for conservation and phylogenetic analyses.

## Materials and methods

**Figure 1.**
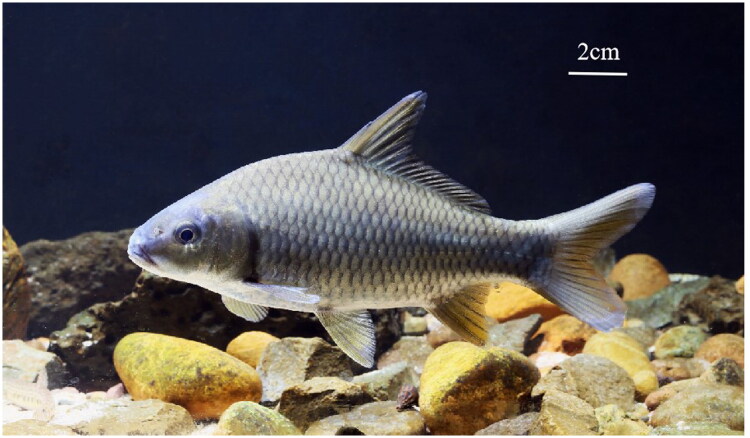
Image of *C. acutidorsalis* from the Hainan Wanquan River, taken by Shen Zhixin.

The sample of *C. acutidorsalis* (Figure 1) was collected from Wanquan river, Qionghai City, Hainan Province (19°11′31.06″ N, 110°56′01.62″ E) and deposited in the Hainan Academy of Ocean and Fisheries (http://www.hnhky.cn/, Qingfeng Zhang, zhangqf@hnhky.cn) under the voucher number HNFF0620101. Total DNA was extracted using an Ezup DNA kit and then sequenced with an Illumina 6000. The genome was assembled using GetOrganelle (Jin et al. 2020). The annotated sequence was submitted to GenBank with the accession number OQ658734.1. The read coverage depth map is shown in Figure S1. A phylogenetic tree was analyzed by software RAxML (Stamatakis 2014), using the following sequences from Genbank: *C. carpio Guilin* (MK291479), *C. carpio Oujiang* (KP993136.1), and *C. carpio Zujiang* (KP993137.1), (Dong et al. 2019); *C. carpio* (AP009047.1), (Ye et al. 2018); *C. acutidorsalis* (KR869145.1), *C. multitaeniata* (KR869144.1), and *C. carpio wananensis* (KF856964.1), (Lin et al. 2021); and *Opsariichthys pachycephalus* (JN673564.1) (Huang et al. 2017).

## Results

**Figure 2.**
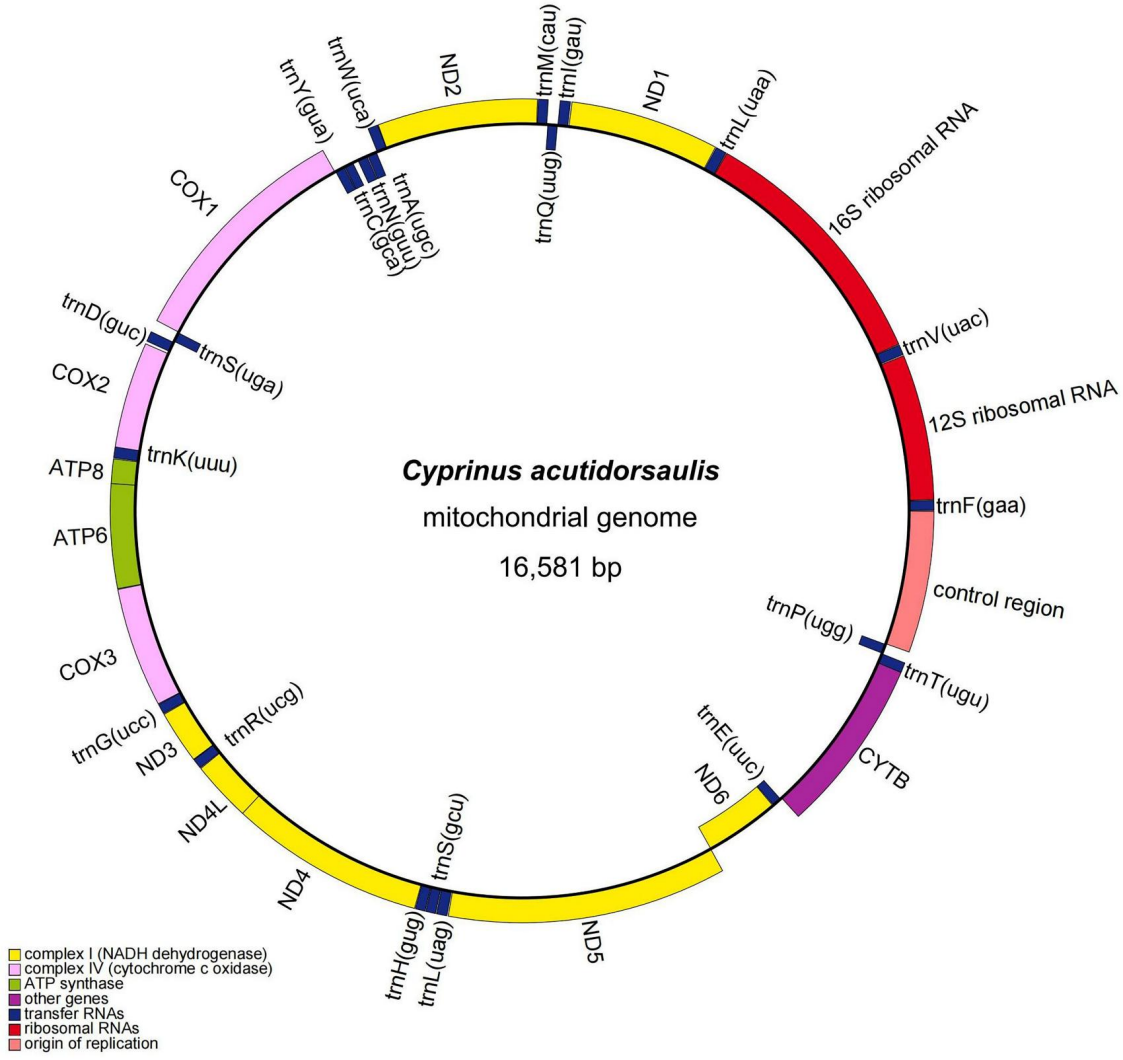
Mitogenome gene map of the *C. acutidorsalis* (OQ658734.1).

**Figure 3.**
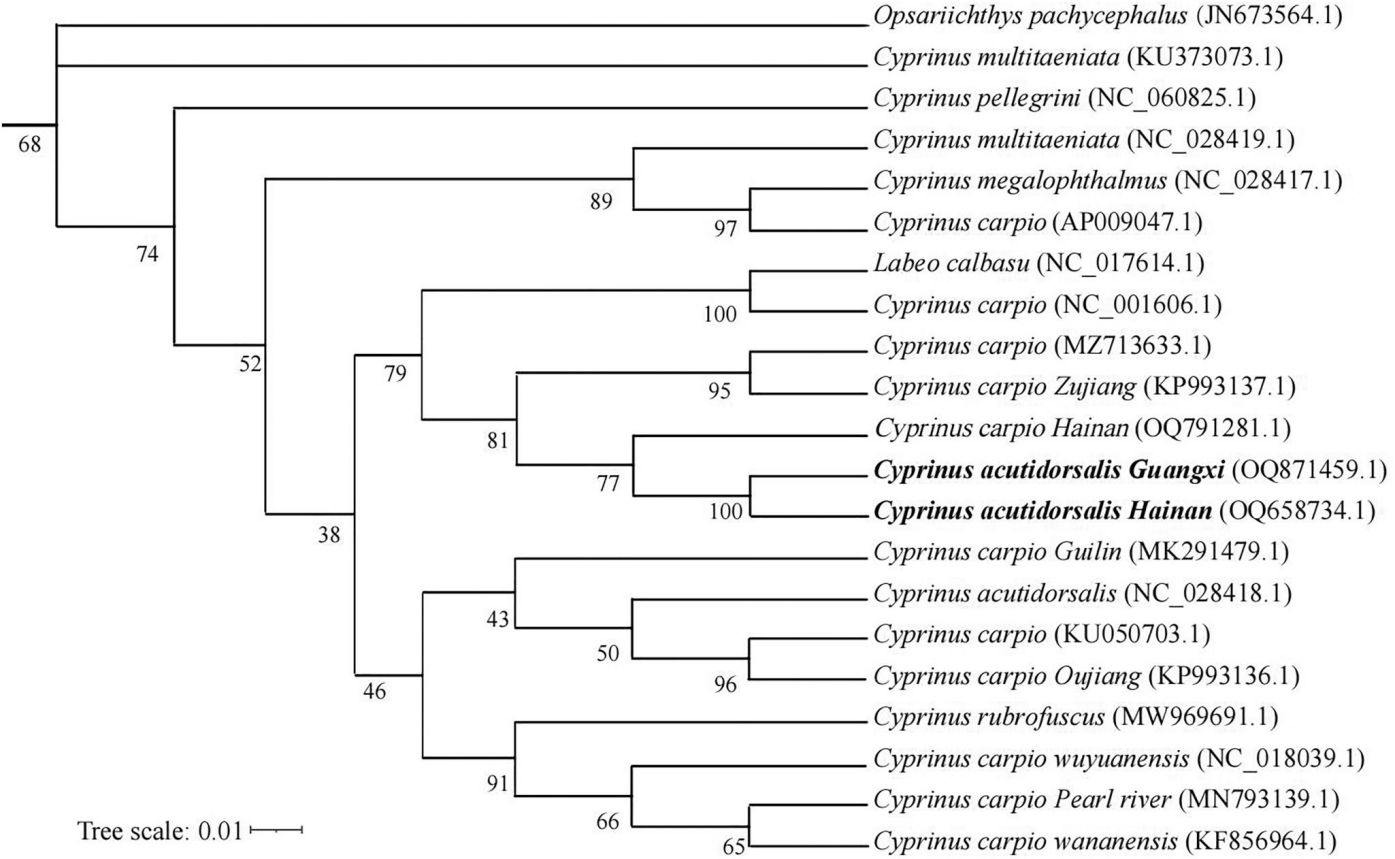
Phylogenetic tree of *C. acutidorsalis* inferred by ML. The position of *C. acutidorsalis* are shown in bold.

The complete circular mitochondrial genome of *C. acutidorsalis* from the Hainan population was 16,581 bp in length (Figure 2), with a base composition of 32.0, 27.5, 24.8 and 15.7% of A, C, T, and G, respectively. The composition showed a slight bias toward A + T content (56.8%), which is consistent with other *Cyprinus* fishes (Ma et al. 2019; Xue et al. 2019). The mitogenome contained 13, 22, and 2 protein-coding genes (PCGs), tRNA, and rRNA-coding genes, respectively, along with a control region. All the PCGs had an ATG start codon, except for *COX1* (GTG) and *ND6* (TTA). Five PCGs (*ND1*, *COX1*, *ATP6*, *ND4L*, and *ND5*) terminated with TAA and eight PCGs (*ND2*, *COX2*, *ATP8*, *COX3*, *ND3*, *ND4*, *ND6*, and *CYTB*) ended with an incomplete stop codon (TA– or T– –). Except for *ND6* and the eight tRNA genes, all other genes were encoded on the heavy strand (H). The Maximum Likelihood (ML) tree (Figure 3) showed that *C. acutidorsalis* from the Hainan population was most related to that of the *C. acutidorsalis* Guangxi population, and the nucleotide sequence divergence of 13 PCGs between the two populations was 0.1%. Interestingly, although *C. acutidorsalis* is endemic, *C. carpio* is widespread worldwide, the interspecific mitogenome sequence divergence between these two species was small (0.3%). This divergence was lower than that of *C. megalophthalmus* (1.8%), the other *Cyprinus* species evaluated in this study.

## Discussion and conclusion

The mitochondrial genome usually offers important molecular markers for species identification; however, the differences were indistinguishable at the mitochondrial level between *C. acutidorsalis* and *C. carpio* in both the Hainan and Guangxi populations due to high sequence similarity. In future studies, it is important to consider further whole-genome analyses, including karyogenic analyses, especially for *C. acutidorsalis.* In addtion, the dorsal fin of *C. acutidorsalis* starts after the ventral fin while it start before the ventral fin in *C. carpio*. Besides, the dorsal fin outer margin of *C. acutidorsalis* is significantly concave, and it is flag-like when unfolded. Taken together, our results serve as a reference for conservation research on *C. acutidorsalis* between these two populations and provide a more comprehensive understanding of the phylogenetic relationships among *Cyprinus* species.

## Supplementary Material

Supplemental Material

Supplemental Material

## Data Availability

The genome sequence data that support the findings of this study are openly available in GenBank (https://www.ncbi.nlm.nih.gov) under the accession number OQ658734.1. The associated **BioProject**, **SRA**, and **Bio-Sample** numbers are PRJNA967109, SRR24424785, and SAMN34576991, respectively.
