# Supplementary figures and images for: Phylogenetic analysis of Cyprinus acutidorsalis (Wang, 1979) from the Hainan population using complete mitochondrial genome
Source: Mitochondrial DNA B Resour. 2024 Jun 24;9(6):841–4. doi: 10.1080/23802359.2024.2323004 (PMC11198120; doi:10.1080/23802359.2024.2323004)

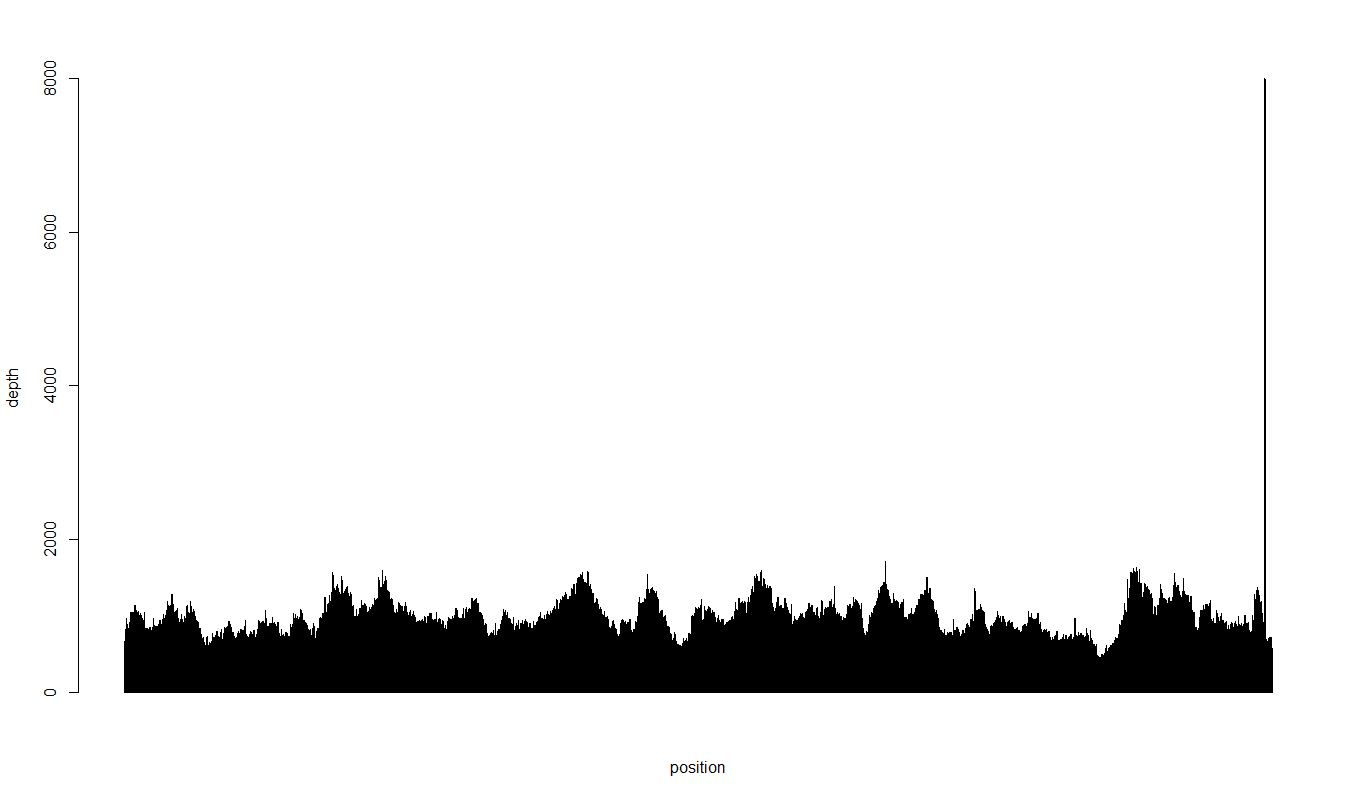

Supplement: Supplemental Material [file TMDN_A_2323004_SM0262.tiff]
